# Supplementary material for: Association between Body Mass Index and Survival Outcome in Metastatic Cancer Patients Treated by Immunotherapy: Analysis of a French Retrospective Cohort
Source: Cancers (Basel). 2021 May 3;13(9):2200. doi: 10.3390/cancers13092200 (PMC8124396; doi:10.3390/cancers13092200)
Supplement: Supplementary file 1 [file cancers-13-02200-s001.zip › cancers-1161226-supplementary.pdf]

Supplementary Data

Table S1 Number of toxicities function of BMI classify in four categories

**Number of toxicities**, n (%)	**BMI <18.5 (n=33)**	**BMI ≥18.5 and <25 (n=146)**	**BMI ≥25 and <30 (n=69)**	**BMI ≥ 30 (n=24)**
No toxicity	21 (63.6)	89 (61)	41 (59.4)	9 (37.5)
One toxicity	6 (18.2)	48 (32.9)	20 (29)	11 (45.8)
Two toxicities	6 (18.2)	9 (6.1)	6 (8.7)	4 (16.7)
Three toxicities	0 (0)	0 (0)	2 (2.9)	0 (0)
